# Supplementary material for: HIVprotI: an integrated web based platform for prediction and design of HIV proteins inhibitors
Source: J Cheminform. 2018 Mar 9;10:12. doi: 10.1186/s13321-018-0266-y (PMC5845081; doi:10.1186/s13321-018-0266-y)
Supplement: Supplementary file 2 — Additional file 2. Source code of HIVProtI web server. [file 13321_2018_266_MOESM2_ESM.zip › HIVprotI_Source-code/hivprotI/weka-3-6-6/doc/weka/classifiers/functions/supportVector/PrecomputedKernelMatrixKernel.html]

PrecomputedKernelMatrixKernel


---


|  |  |  |
| --- | --- | --- |
| **Overview** \| **Package** \| **Class** \| **Tree** \| **Deprecated** \| **Index** \| **Help** \| **Weka's home** | |  |
| **PREV CLASS**   **NEXT CLASS** | **FRAMES**    **NO FRAMES**     **All Classes** |  |
| SUMMARY: NESTED \| FIELD \| CONSTR \| METHOD | DETAIL: FIELD \| CONSTR \| METHOD |  |


---


## weka.classifiers.functions.supportVector Class PrecomputedKernelMatrixKernel

```
java.lang.Object
  weka.classifiers.functions.supportVector.Kernel
      weka.classifiers.functions.supportVector.PrecomputedKernelMatrixKernel
```

**All Implemented Interfaces:**: java.io.Serializable, CapabilitiesHandler, OptionHandler, RevisionHandler

---

``` public class PrecomputedKernelMatrixKernel extends Kernel ```

This kernel is based on a static kernel matrix that is read from a file. Instances must have a single nominal attribute (excluding the class). This attribute must be the first attribute in the file and its values are used to reference rows/columns in the kernel matrix. The second attribute must be the class attribute.


Valid options are:

```
 -D
  Enables debugging output (if available) to be printed.
  (default: off)
```

```
 -no-checks
  Turns off all checks - use with caution!
  (default: checks on)
```

```
 -M <file name>
  The file name of the file that holds the kernel matrix.
  (default: kernelMatrix.matrix)
```

**Version:**
:   $Revision: 5518 $

**Author:**
:   Eibe Frank (eibe@cs.waikato.ac.nz)

**See Also:**: Serialized Form

---

**Constructor Summary**

`PrecomputedKernelMatrixKernel()`


| **Method Summary** | |
| --- | --- |
| `void` | `clean()`             Frees the memory used by the kernel. |
| `double` | `eval(int id1, int id2, Instance inst1)`             Computes the result of the kernel function for two instances. |
| `Capabilities` | `getCapabilities()`             Returns the Capabilities of this kernel. |
| `java.io.File` | `getKernelMatrixFile()`             Gets the file containing the kernel matrix. |
| `java.lang.String[]` | `getOptions()`             Gets the current settings of the Kernel. |
| `java.lang.String` | `getRevision()`             Returns the revision string. |
| `java.lang.String` | `globalInfo()`             Returns a string describing the kernel |
| `java.lang.String` | `kernelMatrixFileTipText()`             Returns the tip text for this property |
| `java.util.Enumeration` | `listOptions()`             Returns an enumeration describing the available options. |
| `int` | `numCacheHits()`             Returns the number of dot product cache hits. |
| `int` | `numEvals()`             Returns the number of kernel evaluation performed. |
| `void` | `setKernelMatrixFile(java.io.File f)`             Sets the file holding the kernel matrix |
| `void` | `setOptions(java.lang.String[] options)`             Parses a given list of options. |
| `java.lang.String` | `toString()`             returns a string representation for the Kernel |

**Methods inherited from class weka.classifiers.functions.supportVector.Kernel**

`buildKernel, checksTurnedOffTipText, debugTipText, forName, getChecksTurnedOff, getDebug, makeCopies, makeCopy, setChecksTurnedOff, setDebug`

**Methods inherited from class java.lang.Object**

`equals, getClass, hashCode, notify, notifyAll, wait, wait, wait`

**Constructor Detail**

### PrecomputedKernelMatrixKernel

```
public PrecomputedKernelMatrixKernel()
```


**Method Detail**

### globalInfo

```
public java.lang.String globalInfo()
```

:   Returns a string describing the kernel

    :   **Specified by:**: `globalInfo` in class `Kernel`
    :   **Returns:**: a description suitable for displaying in the explorer/experimenter gui

---


### listOptions

```
public java.util.Enumeration listOptions()
```

:   Returns an enumeration describing the available options.

    :   **Specified by:**: `listOptions` in interface `OptionHandler` **Overrides:**: `listOptions` in class `Kernel`
    :   **Returns:**: an enumeration of all the available options.

---


### setOptions

```
public void setOptions(java.lang.String[] options)
                throws java.lang.Exception
```

:   Parses a given list of options.


    Valid options are:

    ```
     -D
      Enables debugging output (if available) to be printed.
      (default: off)
    ```

    ```
     -no-checks
      Turns off all checks - use with caution!
      (default: checks on)
    ```

    ```
     -M <file name>
      The file name of the file that holds the kernel matrix.
      (default: kernelMatrix.matrix)
    ```

    :   **Specified by:**: `setOptions` in interface `OptionHandler` **Overrides:**: `setOptions` in class `Kernel`
    :   **Parameters:**: `options` - the list of options as an array of strings **Throws:**: `java.lang.Exception` - if an option is not supported

---


### getOptions

```
public java.lang.String[] getOptions()
```

:   Gets the current settings of the Kernel.

    :   **Specified by:**: `getOptions` in interface `OptionHandler` **Overrides:**: `getOptions` in class `Kernel`
    :   **Returns:**: an array of strings suitable for passing to setOptions

---


### eval

```
public double eval(int id1,
                   int id2,
                   Instance inst1)
            throws java.lang.Exception
```

:   **Description copied from class: `Kernel`**
:   Computes the result of the kernel function for two instances.
    If id1 == -1, eval use inst1 instead of an instance in the dataset.

    :   **Specified by:**: `eval` in class `Kernel`
    :   **Parameters:**: `id1` - the index of instance 1: `id2` - the index of instance 2: `inst1` - the instance 1 object **Returns:**: the dot product **Throws:**: `java.lang.Exception` - if something goes wrong

---


### getCapabilities

```
public Capabilities getCapabilities()
```

:   Returns the Capabilities of this kernel.

    :   **Specified by:**: `getCapabilities` in interface `CapabilitiesHandler` **Overrides:**: `getCapabilities` in class `Kernel`
    :   **Returns:**: the capabilities of this object **See Also:**: `Capabilities`

---


### setKernelMatrixFile

```
public void setKernelMatrixFile(java.io.File f)
```

:   Sets the file holding the kernel matrix

    :   **Parameters:**: `value` - the exponent value

---


### getKernelMatrixFile

```
public java.io.File getKernelMatrixFile()
```

:   Gets the file containing the kernel matrix.

    :   **Returns:**: the exponent value

---


### kernelMatrixFileTipText

```
public java.lang.String kernelMatrixFileTipText()
```

:   Returns the tip text for this property

    :   **Returns:**: tip text for this property suitable for displaying in the explorer/experimenter gui

---


### toString

```
public java.lang.String toString()
```

:   returns a string representation for the Kernel

    :   **Overrides:**: `toString` in class `java.lang.Object`
    :   **Returns:**: a string representaiton of the kernel

---


### clean

```
public void clean()
```

:   Frees the memory used by the kernel.
    (Useful with kernels which use cache.)
    This function is called when the training is done.
    i.e. after that, eval will be called with id1 == -1.

    :   **Specified by:**: `clean` in class `Kernel`

---


### numEvals

```
public int numEvals()
```

:   Returns the number of kernel evaluation performed.

    :   **Specified by:**: `numEvals` in class `Kernel`
    :   **Returns:**: the number of kernel evaluation performed.

---


### numCacheHits

```
public int numCacheHits()
```

:   Returns the number of dot product cache hits.

    :   **Specified by:**: `numCacheHits` in class `Kernel`
    :   **Returns:**: the number of dot product cache hits, or -1 if not supported by this kernel.

---


### getRevision

```
public java.lang.String getRevision()
```

:   Returns the revision string.

    :   **Specified by:**: `getRevision` in interface `RevisionHandler` **Overrides:**: `getRevision` in class `Kernel`
    :   **Returns:**: the revision


---


|  |  |  |
| --- | --- | --- |
| **Overview** \| **Package** \| **Class** \| **Tree** \| **Deprecated** \| **Index** \| **Help** \| **Weka's home** | |  |
| **PREV CLASS**   **NEXT CLASS** | **FRAMES**    **NO FRAMES**     **All Classes** |  |
| SUMMARY: NESTED \| FIELD \| CONSTR \| METHOD | DETAIL: FIELD \| CONSTR \| METHOD |  |


---
